# Association of tramadol with all-cause mortality, cardiovascular diseases, venous thromboembolism, and hip fractures among patients with osteoarthritis: a population-based study

**DOI:** 10.1186/s13075-022-02764-3

**Published:** 2022-04-11

**Authors:** Lingyi Li, Shelby Marozoff, Na Lu, Hui Xie, Jacek A. Kopec, Jolanda Cibere, John M. Esdaile, J. Antonio Aviña-Zubieta

**Affiliations:** 1 Arthritis Research Canada, 230-2238 Yukon Street, Vancouver, BC V5Y 3P2 Canada; 2 Experimental Medicine Program, Faculty of Medicine, University of British Columbia, Vancouver, Canada; 3 Faculty of Health Sciences, Simon Fraser University, Burnaby, Canada; 4 School of Population and Public Health, University of British Columbia, Vancouver, Canada; 5 Division of Rheumatology, Department of Medicine, University of British Columbia, Vancouver, Canada

**Keywords:** Osteoarthritis, Tramadol, Mortality, Cardiovascular diseases, Venous thromboembolism, Hip fractures

## Abstract

**Background:**

The use of tramadol among osteoarthritis (OA) patients has been increasing rapidly around the world, but population-based studies on its safety profile among OA patients are scarce. We sought to determine if tramadol use in OA patients is associated with increased risks of all-cause mortality, cardiovascular diseases (CVD), venous thromboembolism (VTE), and hip fractures compared with commonly prescribed nonsteroidal anti-inflammatory drugs (NSAIDs) or codeine.

**Methods:**

Using administrative health datasets from British Columbia, Canada, we conducted a sequential propensity score-matched cohort study among all OA patients between 2005 and 2013. The tramadol cohort (i.e., tramadol initiation) was matched with four comparator cohorts (i.e., initiation of naproxen, diclofenac, cyclooxygenase-2 [Cox-2] inhibitors, or codeine). Outcomes are all-cause mortality, first-ever CVD, VTE, and hip fractures within the year after the treatment initiation. Patients were followed until they either experienced an event, left the province, or the 1-year follow-up period ended, whichever occurred first. Cox proportional hazard models were used to estimate hazard ratios after adjusting for competing risk of death.

**Results:**

Overall, 100,358 OA patients were included (mean age: 68 years, 63% females). All-cause mortality was higher for tramadol compared to NSAIDs with rate differences (RDs/1000 person-years, 95% CI) ranging from 3.3 (0.0–6.7) to 8.1 (4.9–11.4) and hazard ratios (HRs, 95% CI) ranging from 1.2 (1.0–1.4) to 1.5 (1.3–1.8). For CVD, no differences were observed between tramadol and NSAIDs. Tramadol had a higher risk of VTE compared to diclofenac, with RD/1000 person-years (95% CI) of 2.2 (0.7–3.7) and HR (95% CI) of 1.7 (1.3–2.2). Tramadol also had a higher risk of hip fractures compared to diclofenac and Cox-2 inhibitors with RDs/1000 person-years (95% CI) of 1.9 (0.4–3.4) and 1.7 (0.2–3.3), respectively, and HRs (95% CI) of 1.6 (1.2–2.0) and 1.4 (1.1–1.9), respectively. No differences were observed between tramadol and NSAIDs for all events.

**Conclusions:**

OA patients initiating tramadol have an increased risk of mortality, VTE, and hip fractures within 1 year compared with commonly prescribed NSAIDs, but not with codeine.

**Supplementary Information:**

The online version contains supplementary material available at 10.1186/s13075-022-02764-3.

## Background

Osteoarthritis (OA) is the most common type of arthritis and is recognized as one of the most important health problems in modern industrial societies [1, 2]. In 2017, OA affected 303 million people globally [3]. In 2013, it was the second most costly health condition treated at United States (US) hospitals with a total of $16.5 billion in aggregate hospital costs [4]. OA is associated with cartilage degradation which can lead to pain and decreased mobility [5]. As there is no effective treatment available that can halt OA progression, the main goal of medical therapy for managing OA is to control pain while avoiding therapeutic toxicity [6]. Few safe and effective treatments are available for OA patients. Tramadol, a weak opioid agonist, has been recommended by the 2013 American Academy of Orthopaedic Surgeons guidelines and recommended conditionally by the 2012 American College of Rheumatology guidelines for symptomatic knee OA, along with nonsteroidal anti-inflammatory drugs (NSAIDs) [7, 8]. Thus, the use of tramadol among OA patients has been increasing rapidly around the world. For example, in the US, the prescription of tramadol for the management of knee OA doubled from 5 to 10% between 2003 and 2009 and 44 million tramadol prescriptions were given in 2014 [9, 10]. In the United Kingdom (UK), the prevalence of OA patients with a prescription for tramadol increased from 3 to 10% from 2000 to 2015 [11]. In the province of British Columbia (BC), Canada, tramadol use for OA patients increased steadily from its introduction in 2005 and it has been the second most commonly prescribed opioid agonist since 2008 [12].

As suggested by a recent meta-analysis on the comparative effectiveness of NSAIDs and opioid use for knee OA, there is no statistically significant difference in pain relief between tramadol and NSAIDs among OA patients [13]; however, tramadol is associated with more opioid-related adverse effects, for example, nausea, dizziness, constipation, tiredness, headache, vomiting, and drowsiness [14]. Several studies have compared risks of serious adverse events between tramadol and alternative commonly prescribed analgesics in patients with OA using the Health Improvement Network data that includes 6% of the UK population [11, 15, 16]. These studies showed that tramadol was associated with a significantly higher risk of mortality, myocardial infarction (MI), and hip fractures as compared to commonly prescribed NSAIDs. However, to describe the safety profile of tramadol among OA patients, the results need to be confirmed in a truly population-based sample. This study aimed to determine if tramadol initiation is associated with an increased risk of all-cause mortality, as well as incident cardiovascular diseases (CVD), venous thromboembolism (VTE), and hip fractures compared with other commonly prescribed analgesics for OA using the entire population of the province of BC, Canada.

## Methods

### Data source

Universal healthcare coverage is available for all residents of BC, Canada (population ~ 4.7 million in 2014). Population Data BC captures all provincially funded healthcare services from 1990, including all healthcare professional visits [17], hospitalizations [18], demographic data [19], BC cancer registry [20], and vital statistics [21]. Furthermore, Population Data BC includes the comprehensive prescription drug database PharmaNet [22], which captures all outpatient dispensed medications for all residents since 1996. Numerous population-based studies have been successfully conducted using Population Data BC [23–26].

### Study design and cohort definitions

Using Population Data BC, eligible patients with OA (aged 50 years and older) who received medical care from January 1, 2005, to December 31, 2013, were included. Our case definition of OA consisted of at least two visits to a health professional within 2 years on separate days or one discharge from the hospital with an International Classification of Disease 9th revision code of 715 or International Classification of Disease 10th revision code of M15 to M19. A visit was defined as any service with the exclusion of diagnostic procedures and certain other procedures, such as dialysis/transfusion, anesthesia, obstetrics, or therapeutic radiation. Similar OA case definitions have been used in previous studies in Canada and found to have a positive predictive value varying from 82 to 100% [27, 28]. All OA patients had at least 1 year of continuous enrollment.

We conducted a sequential propensity score-matched cohort study with four comparison cohorts to assess the risk of all-cause mortality, CVD, VTE, and hip fractures between OA patients who received an initial prescription for tramadol and OA patients who received an initial prescription for one of the following medications: naproxen, diclofenac (nonselective NSAIDs), a cyclooxygenase-2 [Cox-2] inhibitor, or codeine (a commonly prescribed weak opioid) from January 1, 2005, to December 31, 2013. Eligible participants were required to have no prescriptions for tramadol or the comparator medication in the year prior to their initial prescription (i.e., the index date). Patients with a history of cancer were excluded. All participants had at least 1 year of follow-up starting from the index date.

### Assessment of outcomes

Outcomes of this study were (1) all-cause mortality, (2) incident CVD (MI or ischemic stroke), (3) VTE (pulmonary embolism [PE] or deep vein thrombosis [DVT]), and (4) hip fractures within the first year following initiation of tramadol or its comparators. Case definitions for each outcome are listed in Supplemental Table 1. Similar case definitions for each outcome condition have been validated by previous studies with positive predictive values ranging between 82 and 96% [29–32].

To identify incident cases, patients with a history of each outcome event of interest prior to the index date were excluded. Patients were followed until the corresponding outcome occurred, they left the province, or the end of the 1-year follow-up period, whichever occurred first.

### Statistical analysis

Calendar years from January 1, 2005, to December 31, 2013, were divided into nine 1-year blocks. Propensity scores were calculated for the initial prescription of tramadol using logistic regression. The variables included in the model were registration start date, socio-demographic factors (i.e., age at the index date and sex), OA duration, comorbidities (myocardial infarction, ischemic heart disease, heart failure, congestive heart failure, peripheral vascular disease, cerebrovascular disease, dementia, chronic obstructive pulmonary disease, rheumatic disorder, chronic kidney disease, peptic ulcer disease, liver disease, diabetes, obesity, hypertension, angina, atrial fibrillation) ever prior to the index date. Comorbidities were identified using International Classification 9th and 10th revision codes. Prescriptions (aspirin, angiotensin-converting enzyme inhibitors, angiotensin receptor blockers, beta blockers, statins, diuretics, fibrates, nitrates, anticoagulants, antidiabetic medicines, other NSAIDs, other opioids, glucocorticoids, serotonin-norepinephrine reuptake inhibitors, selective serotonin reuptake inhibitors, benzodiazepines, other anti-epileptic medications) during the year prior to the index date (yes/no) were from the PharmaNet drug database. The number of healthcare utilizations was counted from the outpatient visits and hospitalizations during the year prior to the index date. We used standardized differences less than 0.10 to define a balance measure of individual covariates before and after propensity score matching [33]. Within each 1-year time block, tramadol users were matched 1:1 to the users of each of the other comparator analgesics using the greedy matching method [34]. In this way, we assembled four comparison groups: tramadol vs. naproxen, tramadol vs. diclofenac, tramadol vs. Cox-2 inhibitors, and tramadol vs. codeine.

We compared the baseline characteristics of the four tramadol cohorts with each of the four comparison cohorts both before and after the propensity score matching. We calculated person-years of follow-up for each patient and the incidence rate for each cohort and plotted cumulative incidence curves of all-cause mortality, CVD, VTE, and hip fractures. We examined the rate difference (RD)/1000 person-years in each outcome between the tramadol cohort with each of the four comparison cohorts using an additive hazard model [35]. The effect estimate generated from this model can be interpreted as the number of excess events attributable to tramadol per 1000 person-years. We compared the rate of each outcome in the tramadol cohort with each of the four comparison cohorts using Cox proportional hazard models adjusted for calendar year. We used the Fine-Gray method [36] to account for the competing risk of death for the event of CVD, VTE, and hip fractures.

All statistical analyses were performed using SAS version 9.4. For all hazard ratios (HRs), we calculated 95% confidence intervals (95% CI).

## Results

As shown in Table 1, OA patients in the tramadol cohorts, in general, were older and had a longer duration of OA, a higher prevalence of comorbidities, a higher use of the majority of other prescriptions, and a higher number of healthcare visits or hospitalization than OA patients in the NSAID cohorts and the codeine cohort before propensity score matching.

**Table 1:** Baseline characteristics of unmatched osteoarthritis patients in a study comparing the association of tramadol and other analgesics with all-cause mortality, myocardial infarction, ischemic stroke, venous thromboembolism, and hip fractures

	Tramadol ***N***=19026	Naproxen ***N***=25793	Standard difference	Tramadol ***N***=19648	Diclofenac ***N***=43588	Standard difference	Tramadol ***N***=25830	Cox-2 ***N***=21786	Standard difference	Tramadol ***N***=7010	Codeine ***N***=28229	Standard difference
Age, mean (SD)	70.0 (10.8)	66.5 (10.1)	0.343	68.2 (10.5)	68.0 (10.4)	0.023	69.2 (11.2)	67.6 (10.7)	0.148	69.0 (10.5)	68.4 (10.5)	0.053
OA duration, mean (SD)	7.2 (6.0)	6.5 (5.5)	0.109	6.8 (5.8)	6.5 (5.3)	0.044	7.1 (6.0)	6.1 (5.7)	0.175	6.6 (5.9)	5.9 (5.3)	0.114
Male (%)	36.7	41.3	0.095	39.2	37.6	0.034	39.6	39.1	0.012	27.1	39.9	0.273
**Comorbidity (%)**												
Myocardial infarction	10.1	6.5	0.131	9.5	7.3	0.078	11.0	6.3	0.166	7.1	6.5	0.025
Ischemic heart disease	19.4	16.3	0.081	18.0	17.8	0.003	19.9	16.0	0.1	15.0	14.6	0.012
Heart failure	5.8	3.0	0.139	4.8	3.7	0.054	5.8	2.9	0.143	3.9	3.2	0.042
Congestive heart failure	14.0	8.3	0.181	12.4	9.9	0.081	14.5	8.6	0.183	10.7	8.9	0.058
Peripheral vascular disease	2.5	1.3	0.083	2.3	1.6	0.047	2.6	1.4	0.085	1.6	1.2	0.033
Cerebrovascular disease	15.3	9.4	0.178	13.7	11.4	0.071	15.5	10.3	0.157	12.7	10.4	0.072
Dementia	4.6	3.0	0.085	3.9	3.0	0.052	4.8	3.1	0.084	3.4	4.0	0.029
Chronic obstructive pulmonary disease	49.7	45.2	0.09	50.4	47.2	0.065	52.8	46.3	0.129	45.0	36.2	0.181
Rheumatic disorder	10.4	7.7	0.093	9.3	8.1	0.043	9.3	7.4	0.069	9.8	6.2	0.133
Chronic kidney disease	11.4	5.4	0.218	10.2	6.2	0.145	11.5	5.7	0.207	8.8	6.0	0.108
Peptic ulcer disease	13.9	12.6	0.039	13.1	12.2	0.025	13.4	12.5	0.027	12.6	8.5	0.133
Liver disease	1.4	1.2	0.013	1.5	1.2	0.023	1.7	1.1	0.053	1.0	0.8	0.021
Diabetes	30.1	28.2	0.042	30.5	29.7	0.018	32.7	29.9	0.061	26.3	25.5	0.019
Obesity	14.6	12.8	0.053	15.4	13.2	0.061	15.8	13.6	0.061	14.9	10.3	0.14
Hypertension	74.4	65.5	0.195	71.6	68.8	0.062	73.5	67.5	0.134	70.2	65.9	0.092
Angina	33.4	26.7	0.148	31.7	28.9	0.061	33.7	27.0	0.145	29.5	24.5	0.113
Atrial fibrillation	10.3	4.4	0.228	8.8	6.0	0.105	9.8	4.7	0.2	7.8	6.0	0.073
**Medication (%)**												
Aspirin	5.2	4.1	0.052	5.2	4.5	0.029	6.1	4.6	0.069	3.5	5.0	0.072
ACE inhibitors	12.4	13.1	0.022	11.1	15.4	0.127	11.6	13.1	0.044	10.5	14.5	0.124
Angiotensin receptor blocker	1.5	1.5	0.007	1.4	1.7	0.026	1.6	1.6	0.001	1.0	1.5	0.048
Calcium channel blockers	4.6	2.9	0.088	3.7	3.6	0.004	4.3	2.9	0.077	3.8	3.1	0.04
Beta blocker	19.3	13.4	0.159	18.2	15.7	0.067	19.3	13.5	0.156	16.4	14.9	0.04
Statin	23.3	20.7	0.061	21.6	23.4	0.044	22.6	21.7	0.021	20.1	20.4	0.008
Diuretics	25.2	18.6	0.161	23.4	21.9	0.036	24.9	19.6	0.129	23.4	20.6	0.066
Fibrates	0.9	1.0	0.017	0.9	1.1	0.018	0.9	0.9	0.006	1.2	0.8	0.032
Nitrates	5.9	3.9	0.092	5.4	4.7	0.032	6.3	3.5	0.129	4.7	3.8	0.047
Anticoagulants	13.5	3.6	0.358	11.9	5.6	0.224	11.8	8.0	0.127	11.2	9.4	0.057
Antidiabetic medicine	10.3	9.9	0.015	10.8	10.7	0.004	12.0	10.3	0.055	9.2	8.8	0.016
Other NSAIDs	34.2	27.4	0.147	31.0	32.1	0.023	35.7	39.6	0.082	38.7	32.9	0.122
Other opioids	39.6	30.7	0.188	40.1	32.4	0.163	42.1	34.7	0.154	12.8	4.8	0.288
Glucocorticoids	14.3	9.5	0.149	13.2	11.4	0.056	14.4	12.9	0.042	12.8	9.1	0.119
SNRIs	4.0	3.0	0.052	4.0	3.2	0.043	4.1	3.2	0.048	3.6	2.2	0.082
SSRIs	11.3	9.2	0.069	11.3	9.6	0.054	11.5	9.9	0.052	10.8	7.7	0.106
Benzodiazepines	20.0	15.8	0.111	19.3	17.4	0.047	20.3	16.5	0.098	17.8	12.4	0.151
Other anti-epileptic medication	12.5	6.8	0.193	11.9	6.9	0.172	12.7	8.5	0.138	11.3	5.0	0.234
**Healthcare utilization, mean (SD)**												
Outpatient visits	23.6 (17.1)	17.1 (13.5)	0.425	22.4 (16.3)	19.2 (14.5)	0.207	23.6 (17.1)	19.0 (14.0)	0.297	21.5 (15.1)	17.5 (13.7)	0.277
Hospitalizations	0.8 (1.0)	0.3 (0.7)	0.566	0.8 (1.0)	0.4 (0.8)	0.46	0.8 (1.0)	0.4 (0.8)	0.44	0.8 (0.9)	0.5 (0.8)	0.243

*Abbreviations*: *OA* osteoarthritis, *ACE* angiotensin-converting enzyme, *SNRIs* serotonin-norepinephrine reuptake inhibitors, *SSRIs* selective serotonin reuptake inhibitors

After propensity score matching, 100,358 patients with OA were included (mean age of 68 years, 63% were females). Of the matched OA patients, 12,269 were included in the naproxen cohort, 15,749 in the diclofenac cohort, 15,410 in the Cox-2 inhibitor cohort, and 6751 in the codeine cohort. The baseline characteristics between the matched cohorts were well balanced, with all standardized differences less than 0.10 (Table 2).

**Table 2:** Baseline characteristics of propensity score-matched osteoarthritis patients in a study comparing the association of tramadol and other analgesics with all-cause mortality, myocardial infarction, ischemic stroke, venous thromboembolism, and hip fractures

	Tramadol ***N***=12269	Naproxen ***N***=12269	Standard difference	Tramadol ***N***=15749	Diclofenac ***N***=15749	Standard difference	Tramadol ***N***=15410	Cox-2 ***N***=15410	Standard difference	Tramadol ***N***=6751	Codeine ***N***=6751	Standard difference
Age, mean (SD)	68.5 (10.7)	69.0 (10.3)	0.049	68.1 (10.6)	68.2 (10.5)	0.006	68.1 (11.1)	68.1 (10.8)	0.005	68.9 (10.4)	69.2 (10.5)	0.025
OA duration, mean (SD)	7.0 (5.8)	7.0 (5.8)	0.004	6.9 (5.8)	6.9 (5.6)	0.001	6.6 (5.7)	6.6 (6.0)	0.009	6.5 (5.9)	6.5 (5.7)	0.005
Male (%)	38.4	36.5	0.04	38.2	38.2	0.001	39.1	38.7	0.009	28.1	28.1	0.001
**Comorbidity (%)**												
Myocardial infarction	8.2	8.1	0.005	8.7	8.7	<0.001	7.8	7.5	0.012	6.8	7.3	0.017
Ischemic heart disease	18.2	17.8	0.012	17.8	17.7	0.004	17.5	16.8	0.018	14.7	15.1	0.013
Heart failure	4.3	4.0	0.017	4.3	4.3	<0.001	3.8	3.5	0.018	3.6	4.0	0.019
Congestive heart failure	11.2	10.9	0.01	11.8	11.5	0.01	10.3	10.0	0.009	10.3	10.5	0.007
Peripheral vascular disease	1.9	1.8	0.01	2.1	2.1	0.005	1.7	1.7	0.005	1.5	1.5	0.006
Cerebrovascular disease	12.3	12.9	0.017	13.0	13.2	0.007	12.0	12.0	0.002	12.2	12.2	0.002
Dementia	4.2	4.0	0.008	3.8	3.9	0.007	3.8	3.7	0.004	3.5	3.9	0.024
Chronic obstructive pulmonary disease	48.2	48.7	0.011	50.4	50.8	0.006	49.2	50	0.015	44	43.9	0.003
Rheumatic disorder	9.3	9.7	0.016	9.1	9.3	0.006	8.4	8.2	0.006	9.2	9.2	<0.001
Chronic kidney disease	8.5	8.3	0.004	9.2	9.2	0.002	7.5	7.2	0.013	8.5	8.8	0.009
Peptic ulcer disease	13.6	13.4	0.005	13.0	13.1	0.003	13.0	12.7	0.007	11.9	11.8	0.004
Liver disease	1.3	1.3	0.002	1.4	1.4	0.007	1.3	1.3	0.001	1.0	0.8	0.014
Diabetes	29.7	28.8	0.02	31.0	30.7	0.005	31.1	30.7	0.008	26	26.2	0.006
Obesity	13.8	13.9	0.004	15.0	15.4	0.011	14.4	14.9	0.014	14.1	13.8	0.008
Hypertension	71	72.5	0.033	71.6	72	0.009	70.0	70.3	0.007	69.8	69.7	<0.001
Angina	30.5	31.5	0.022	31.6	31.7	0.002	29.8	29.6	0.003	28.4	29.3	0.02
Atrial fibrillation	6.8	6.3	0.019	7.7	7.7	0.001	6.1	5.8	0.014	7.6	7.5	0.002
**Medication (%)**												
Aspirin	5.0	4.8	0.01	5.3	5.4	0.005	5.2	4.9	0.009	3.5	3.8	0.013
ACE inhibitors	12.7	12.9	0.006	11.5	12	0.015	12.6	12.4	0.007	10.3	10.2	0.004
Angiotensin receptor blocker	1.4	1.4	0.002	1.4	1.5	0.003	1.6	1.5	0.001	1.0	1.1	0.007
Calcium channel blockers	3.7	3.8	0.008	3.5	3.5	0.001	3.3	3.3	0.001	3.6	3.8	0.013
Beta blocker	16.4	16.5	0.002	17.3	17.5	0.005	15.4	15.3	0.002	15.9	16.4	0.014
Statin	22.5	23.0	0.014	22.9	23.3	0.009	22.3	22.2	0.001	19.8	20.1	0.007
Diuretics	22.0	22.7	0.018	23.4	23.7	0.007	21.0	21.3	0.006	22.5	22.7	0.004
Fibrates	0.9	0.9	0.002	0.9	1.0	0.009	0.9	0.9	0.001	1.1	1.1	0.003
Nitrates	5.0	4.7	0.012	5.3	5.1	0.005	4.3	4.1	0.01	4.5	4.8	0.014
Anticoagulants	7.1	6.2	0.038	9.7	9	0.026	9.1	9.3	0.005	10.9	10.9	0.001
Antidiabetic medicine	10.4	10.2	0.007	11.2	11.3	0.004	11.1	10.8	0.009	9.0	9.1	0.002
Other NSAIDs	32.3	33.8	0.033	31.9	32.6	0.014	39.8	38.4	0.028	38	37.9	0.002
Other opioids	36.8	37.9	0.023	39.7	40.5	0.018	38.7	39.2	0.012	10.9	10.7	0.008
Glucocorticoids	12.7	12.8	0.005	13.2	13.6	0.009	13.8	13.8	0.002	12.2	12.2	0.001
SNRIs	3.6	3.7	0.005	4.0	4.1	0.001	3.6	3.5	0.005	3.3	3.3	0.004
SSRIs	10.7	11.0	0.01	11.4	11.6	0.006	10.7	10.6	0.004	10.1	9.9	0.006
Benzodiazepines	18.9	19.0	0.001	19.6	20.2	0.016	18.3	18.7	0.01	16.7	17.3	0.016
Other anti-epileptic medication	10.6	10.6	0.001	11.6	11.8	0.005	10.8	10.6	0.006	10.0	9.7	0.008
**Healthcare utilization, mean (SD)**												
Outpatient visits	20.4 (14.4)	20.1 (15.1)	0.021	21.5 (15.5)	21.5 (16.2)	0.001	20.8 (14.9)	20.3 (14.9)	0.033	20.7 (14.1)	21.0 (16.5)	0.019
Hospitalizations	0.5 (0.8)	0.5 (0.9)	0.052	0.6 (0.9)	0.6 (1.0)	0.021	0.5 (0.8)	0.5 (0.8)	0.028	0.7 (0.8)	0.7 (1.0)	0.011

*Abbreviations*: *OA* osteoarthritis, *ACE* angiotensin-converting enzyme, *SNRIs* serotonin-norepinephrine reuptake inhibitors, *SSRIs* selective serotonin reuptake inhibitors

**Fig. 1:**
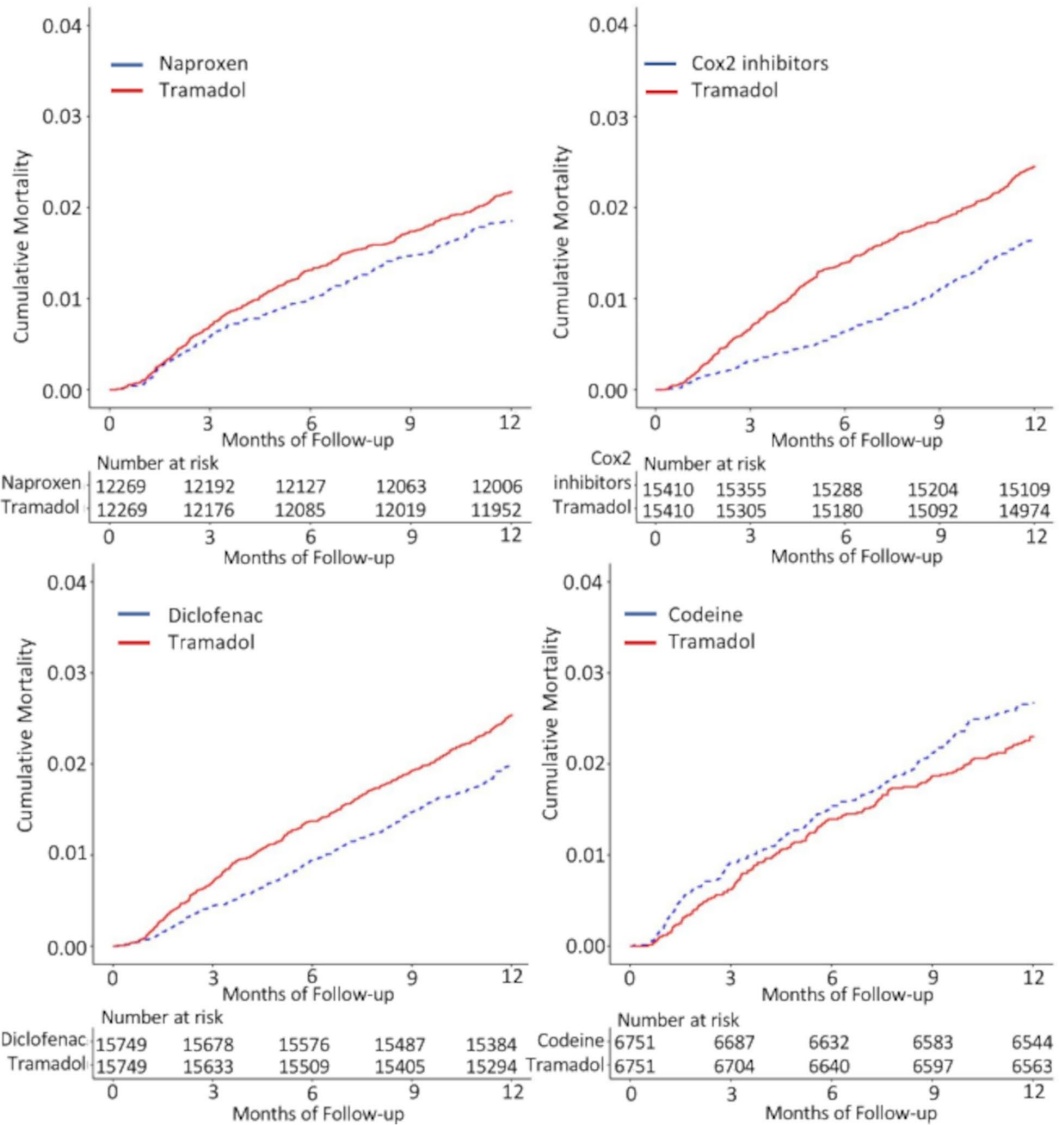
Cumulative incidence of death for propensity score-matched cohorts of osteoarthritis patients with initial prescription of tramadol compared with naproxen, diclofenac, Cox-2 inhibitors, and codeine

Tramadol had a higher all-cause mortality when compared with all NSAIDs, but not with codeine (Table 3). The RDs/1000 person-years (95% CI) comparing tramadol with each comparator were 3.3 (0.0–6.7) for naproxen, 5.6 (2.3–9.0) for diclofenac, 8.1 (4.9–11.4) for Cox-2 inhibitors, and −3.8 (−9.2–1.5) for codeine. The HRs (95% CI) comparing tramadol with each comparator were 1.2 (1.0–1.4) for naproxen, 1.3 (1.1–1.5) for diclofenac, 1.5 (1.3–1.8) for Cox-2 inhibitors, and 0.9 (0.7–1.1) for codeine (Table 3, Fig. 1).

**Table 3:** All-cause mortality within 1 year among patients initiating tramadol compared with other propensity score-matched analgesics among patients with OA

	Group 1		Group 2		Group 3		Group 4	
	Tramadol cohort (***n***=12269)	Naproxen cohort (***n***=12269)	Tramadol cohort (***n***=15749)	Diclofenac cohort (***n***=15749)	Tramadol cohort (***n***=15410)	Cox-2 inhibitors cohort (***n***=15410)	Tramadol cohort (***n***=6751)	Codeine cohort (***n***=6751)
Event (*n*)	266	227	399	313	377	255	155	180
Mean follow-up (years)	0.99	0.99	0.99	0.99	0.99	0.99	0.99	0.98
Rate, per 1000 PY	21.7	18.5	25.3	19.9	24.5	16.6	23.0	26.7
RD (95% CI), per 1000 PY	3.3 (0.0–6.7)	1.0 (ref)	5.6 (2.3–9.0)	1.0 (ref)	8.1 (4.9–11.4)	1.0 (ref)	−3.8 (−9.2–1.5)	1.0 (ref)
HR (95% CI)	1.2 (1.0–1.4)	1.0 (ref)	1.3 (1.1–1.5)	1.0 (ref)	1.5 (1.3–1.8)	1.0 (ref)	0.9 (0.7–1.1)	1.0 (ref)

*Abbreviations*: *OA* osteoarthritis, *PY* person-years, *RD* rate difference, *HR* hazard ratio

**Fig. 2:**
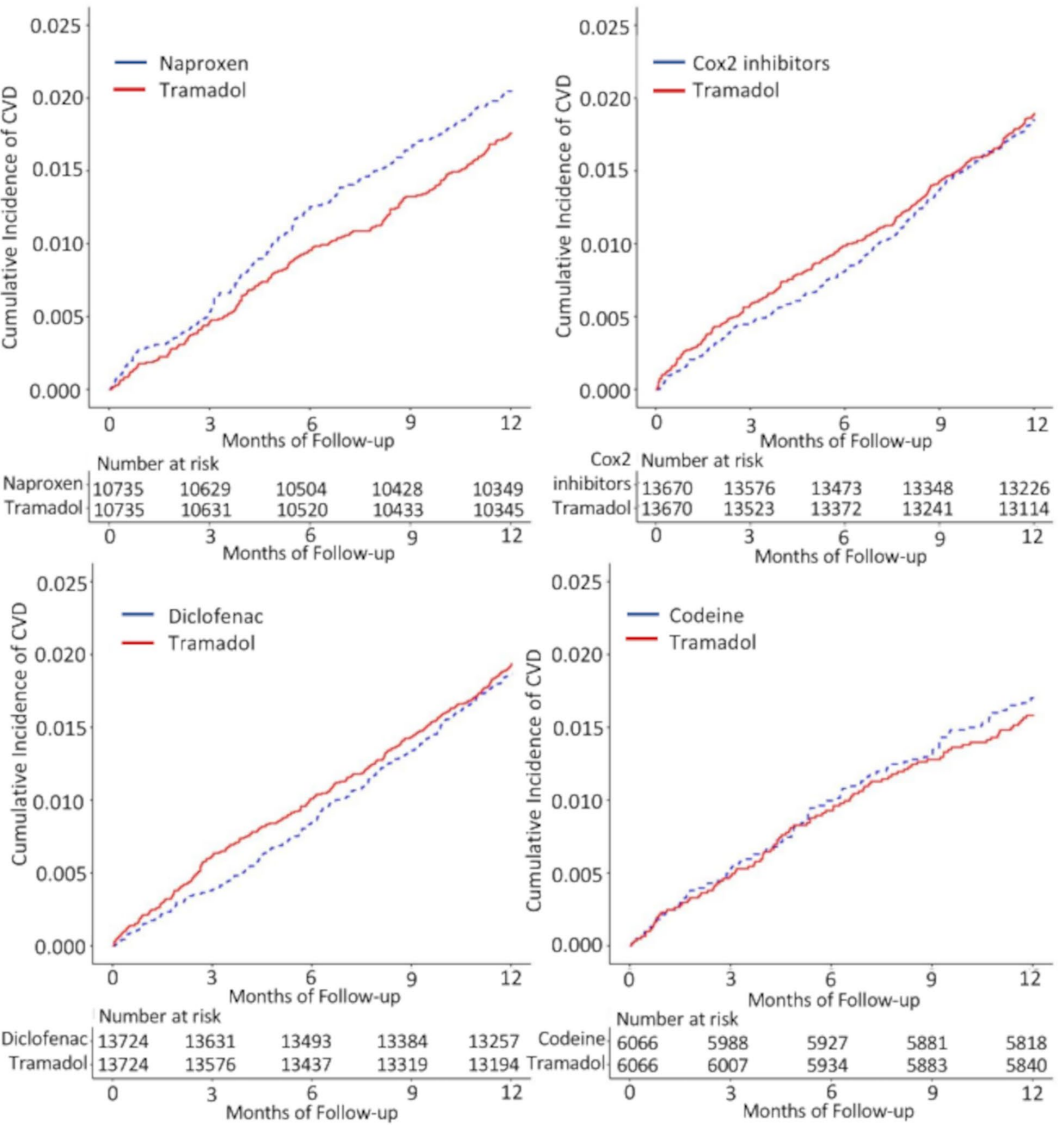
Cumulative incidence of cardiovascular diseases for propensity score-matched cohorts of osteoarthritis patients with initial prescription of tramadol compared with naproxen, diclofenac, Cox-2 inhibitors, and codeine. CVD, cardiovascular diseases

No association between tramadol and CVD (MI or ischemic stroke) was observed when compared with naproxen, diclofenac, Cox-2 inhibitors, and codeine (Table 4). The RDs/1000 person-years (95% CI) of CVD comparing tramadol with each comparator were −2.9 (−6.7–0.7) for naproxen, 0.7 (−2.6–4.0) for diclofenac, 0.5 (−2.8−3.8) for Cox-2 inhibitors, and −1.2 (−5.8–3.4) for codeine. The HRs (95% CI) of CVD comparing tramadol with each comparator were 0.9 (0.7–1.0) for naproxen, 1.0 (0.9–1.2) for diclofenac, 1.0 (0.9–1.2) for Cox-2 inhibitors, and 0.9 (0.8–1.1) for codeine (Table 4, Fig. 2). Similar results were seen in the subgroups — MI and ischemic stroke.

**Table 4:** Cardiovascular disease (myocardial infarction or ischemic stroke) risk within 1 year among patients initiating tramadol compared with other propensity score-matched analgesics among patients with OA

	Group 1		Group 2		Group 3		Group 4	
	Tramadol cohort (***n***=10735)	Naproxen cohort (***n***=10735)	Tramadol cohort (***n***=13724)	Diclofenac cohort (***n***=13724)	Tramadol cohort (***n***=13670)	Cox-2 inhibitors cohort (***n***=13670)	Tramadol cohort (***n***=6066)	Codeine cohort (***n***=6066)
**Cardiovascular diseases**								
Event (*n*)	187	218	263	254	256	251	95	102
Mean follow-up (years)	0.98	0.98	0.98	0.98	0.98	0.98	0.98	0.98
Rate, per 1000 PY	17.4	20.3	19.2	18.5	18.7	18.4	15.7	16.8
RD (95% CI), per 1000 PY	−2.9 (−6.7–0.7)	1.0 (ref)	0.7 (−2.6–4.0)	1.0 (ref)	0.5 (−2.8–3.8)	1.0 (ref)	−1.2 (−5.8–3.4)	1.0 (ref)
HR (95% CI)	0.9 (0.7–1.0)	1.0 (ref)	1.0 (0.9–1.2)	1.0 (ref)	1.0 (0.9–1.2)	1.0 (ref)	0.9 (0.8–1.1)	1.0 (ref)
**Myocardial infarction**								
Event (*n*)	114	135	163	179	155	132	51	55
Mean follow-up (years)	0.98	0.98	0.98	0.99	0.98	0.99	0.98	0.98
Rate, per 1000 PY	10.2	12.1	11.4	12.5	11.0	9.3	8.2	8.8
RD (95% CI), per 1000 PY	−1.9 (−4.7–0.9)	1.0 (ref)	−1.1 (−3.7–1.5)	1.0 (ref)	1.7 (−0.7–4.1)	1.0 (ref)	−0.6 (−3.9–2.6)	1.0 (ref)
HR (95% CI)	0.9 (0.7–1.0)	1.0 (ref)	0.9 (0.8–1.1)	1.0 (ref)	1.2 (1.0–1.4)	1.0 (ref)	0.9 (0.7–1.2)	1.0 (ref)
**Ischemic stroke**								
Event (*n*)	101	97	117	105	128	135	50	57
Mean follow-up (years)	0.98	0.99	0.98	0.99	0.98	0.99	0.95	0.95
Rate, per 1000 PY	9.6	9.2	8.7	7.8	9.6	10.1	8.4	9.6
RD (95% CI), per 1000 PY	0.4 (−2.3–3.1)	1.0 (ref)	0.9 (−1.3–3.1)	1.0 (ref)	−0.5 (−2.9–1.9)	1.0 (ref)	−1.2 (−4.7–2.2)	1.0 (ref)
HR (95% CI)	1.0 (0.8–1.2)	1.0 (ref)	1.1 (0.9–1.3)	1.0 (ref)	1.0 (0.8–1.1)	1.0 (ref)	0.9 (0.7–1.1)	1.0 (ref)

*Abbreviations*: *OA* osteoarthritis, *PY* person-years, *RD* rate difference, *HR* hazard ratio

**Fig. 3:**
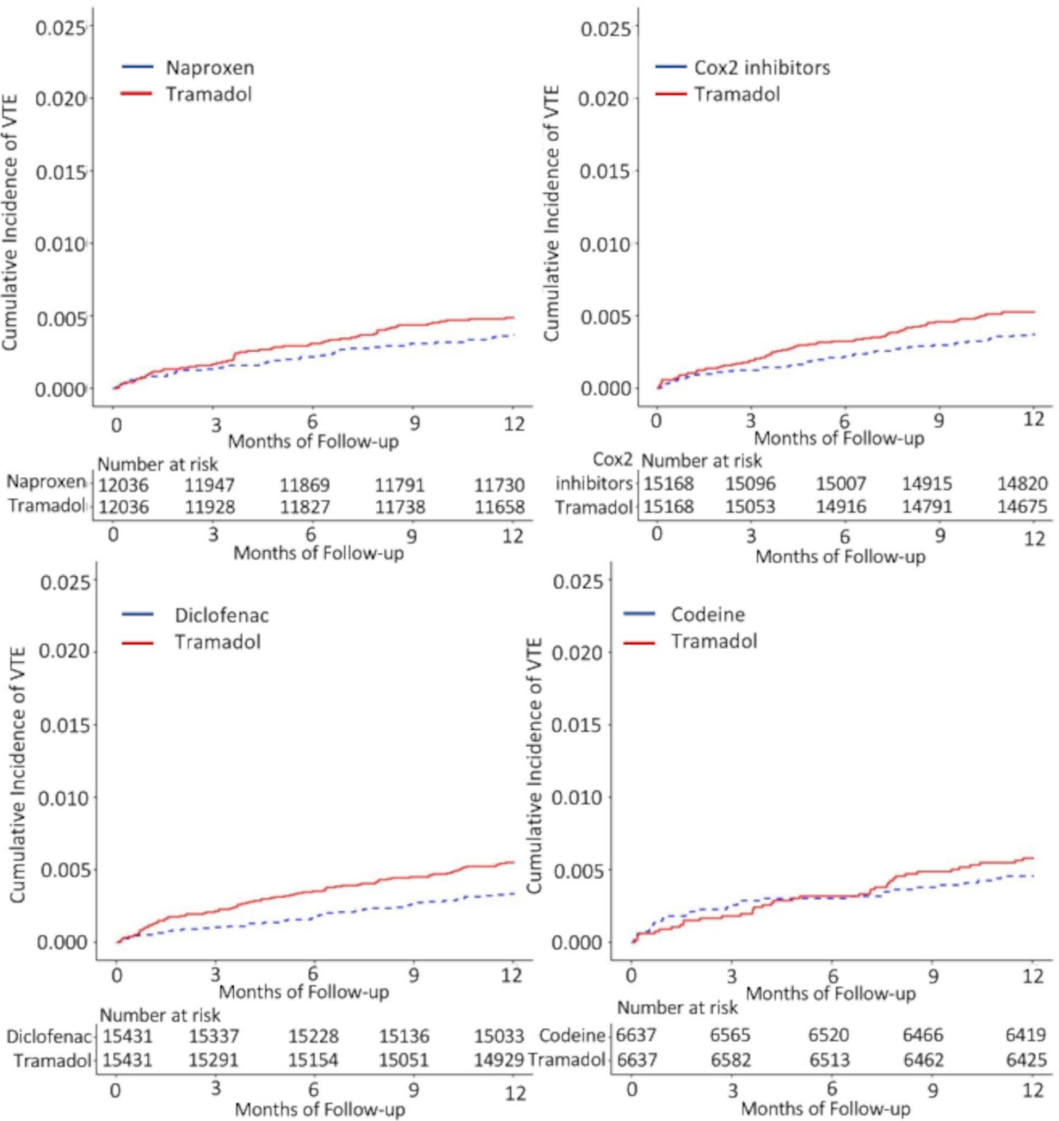
Cumulative incidence of venous thromboembolism for propensity score-matched cohorts of osteoarthritis patients with initial prescription of tramadol compared with naproxen, diclofenac, Cox-2 inhibitors, and codeine. VTE, venous thromboembolism

Tramadol had an association with VTE when compared with diclofenac (Table 5). The RDs/1000 person-years (95% CI) of VTE comparing tramadol with each comparator were 1.2 (−0.4–2.9) for naproxen, 2.2 (0.7–3.7) for diclofenac, 1.4 (−0.1–2.9) for Cox-2 inhibitors, and 1.2 (−1.2–3.7) for codeine. The HRs (95% CI) of VTE comparing tramadol with each comparator were 1.3 (1.0–1.7) for naproxen, 1.7 (1.3–2.2) for diclofenac, 1.4 (1.1–1.8) for Cox-2 inhibitors, and 1.3 (0.9–1.8) for codeine (Table 5, Fig. 3). For PE, we did not see a difference between tramadol with each outcome. For DVT, the RDs/1000 person-years (95% CI) ranged from 1.2 (0.0–2.4) to 1.5 (0.3–2.7) and HRs (95% CI) ranged from 1.5 (1.1–2.0) to 2.0 (1.4–2.8).

**Table 5:** Venous thromboembolism (pulmonary embolism or deep vein thrombosis) risk within 1 year among patients initiating tramadol compared with other propensity score-matched analgesics among patients with OA

	Group 1		Group 2		Group 3		Group 4	
	Tramadol cohort (***n***=12036)	Naproxen cohort (***n***=12036)	Tramadol cohort (***n***=15431)	Diclofenac cohort (***n***=15431)	Tramadol cohort (***n***=15168)	Cox-2 inhibitors cohort (***n***=15168)	Tramadol cohort (***n***=6637)	Codeine cohort (***n***=6637)
**Venous thromboembolism**								
Event (*n*)	58	44	84	51	79	56	38	30
Mean follow-up (years)	0.98	0.99	0.98	0.99	0.98	0.99	0.98	0.98
Rate, per 1000 PY	4.8	3.7	5.4	3.3	5.2	3.7	5.7	4.5
RD (95% CI), per 1000 PY	1.2 (−0.4−2.9)	1.0 (ref)	2.2 (0.7−3.7)	1.0 (ref)	1.4 (−0.1−2.9)	1.0 (ref)	1.2 (−1.2−3.7)	1.0 (ref)
HR (95% CI)	1.3 (1.0−1.7)	1.0 (ref)	1.7 (1.3−2.2)	1.0 (ref)	1.4 (1.1−1.8)	1.0 (ref)	1.3 (0.9−1.8)	1.0 (ref)
**Pulmonary embolism**								
Event (*n*)	30	27	37	31	33	24	17	15
Mean follow-up (years)	0.98	0.99	0.98	0.99	0.99	0.99	0.98	0.98
Rate, per 1000 PY	2.5	2.2	2.4	2.0	2.2	1.6	2.6	2.3
RD (95% CI), per 1000 PY	0.3 (−0.9−1.5)	1.0 (ref)	0.4 (−0.7−1.5)	1.0 (ref)	0.6 (−0.3−1.6)	1.0 (ref)	0.3 (−1.4−2.0)	1.0 (ref)
HR (95% CI)	1.1 (0.8−1.6)	1.0 (ref)	1.2 (0.9−1.7)	1.0 (ref)	1.4 (0.9−2.0)	1.0 (ref)	1.1 (0.7−1.9)	1.0 (ref)
**Deep vein thrombosis**								
Event (*n*)	33	27	54	27	53	35	24	17
Mean follow-up (years)	0.98	0.99	0.98	0.99	0.98	0.99	0.98	0.98
Rate, per 1000 PY	2.7	2.2	3.5	1.8	3.5	2.3	3.6	2.6
RD (95% CI), per 1000 PY	0.5 (−0.7−1.8)	1.0 (ref)	1.5 (0.3−2.7)	1.0 (ref)	1.2 (0.0−2.4)	1.0 (ref)	1.1 (−0.8−3.0)	1.0 (ref)
HR (95% CI)	1.2 (0.9−1.8)	1.0 (ref)	2.0 (1.4−2.8)	1.0 (ref)	1.5 (1.1−2.0)	1.0 (ref)	1.4 (0.9−2.2)	1.0 (ref)

*Abbreviations*: *OA* osteoarthritis, *PY* person-years, *RD* rate difference, *HR* hazard ratio

**Fig. 4:**
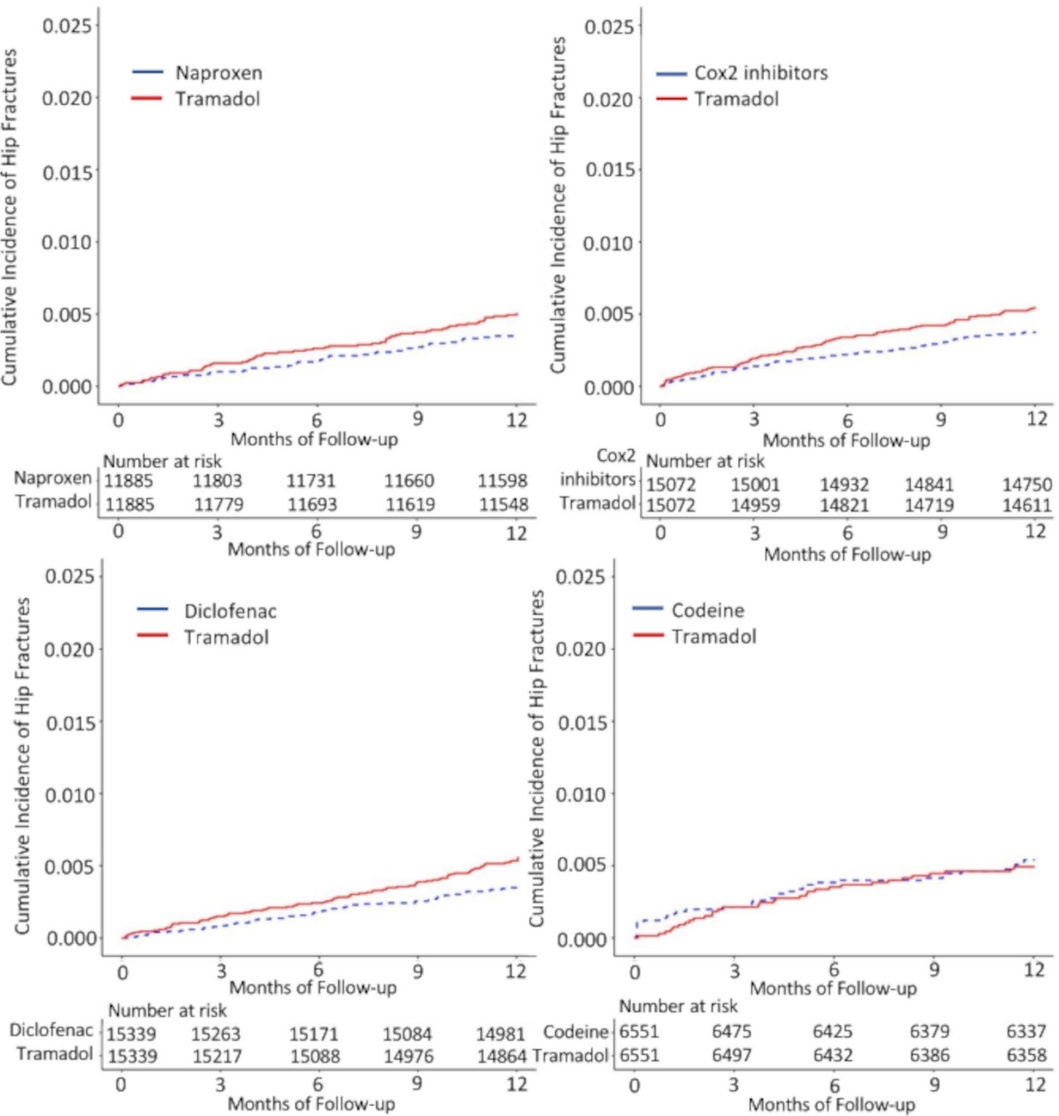
Cumulative incidence of hip fracture for propensity score-matched cohorts of osteoarthritis patients with initial prescription of tramadol compared with naproxen, diclofenac, Cox-2 inhibitors, and codeine

Tramadol had an association with hip fractures when compared with diclofenac and Cox-2 inhibitors, but not with naproxen and codeine (Table 6). The RDs/1000 person-years (95% CI) of hip fractures comparing tramadol with each comparator were 1.5 (−0.2–3.1) for naproxen, 1.9 (0.4–3.4) for diclofenac, 1.7 (0.2–3.3) for Cox-2 inhibitors, and −0.4 (−3.0–2.1) for codeine. The HRs (95% CI) of hip fractures comparing tramadol with each comparator were 1.4 (1.1–1.9) for naproxen, 1.6 (1.2–2.0) for diclofenac, 1.4 (1.1–1.8) for Cox-2 inhibitors, and 0.9 (0.7–1.3) for codeine (Table 6, Fig. 4).

**Table 6:** Hip fracture risk within 1 year among patients initiating tramadol compared with other propensity score-matched analgesics among patients with OA

	Group 1		Group 2		Group 3		Group 4	
	Tramadol cohort (***n***=11885)	Naproxen cohort (***n***=11885)	Tramadol cohort (***n***=15339)	Diclofenac cohort (***n***=15339)	Tramadol cohort (***n***=15072)	Cox-2 inhibitors cohort (***n***=15072)	Tramadol cohort (***n***=6551)	Codeine cohort (***n***=6551)
Event (*n*)	59	42	82	53	81	56	32	35
Mean follow-up (years)	0.98	0.99	0.98	0.99	0.98	0.99	0.98	0.98
Rate, per 1000 PY	5.0	3.5	5.4	3.5	5.4	3.7	4.9	5.3
RD (95% CI), per 1000 PY	1.5 (−0.2−3.1)	1.0 (ref)	1.9 (0.4−3.4)	1.0 (ref)	1.7 (0.2−3.3)	1.0 (ref)	−0.4 (−3.0−2.1)	1.0 (ref)
HR (95% CI)	1.4 (1.1−1.9)	1.0 (ref)	1.6 (1.2−2.0)	1.0 (ref)	1.4 (1.1−1.8)	1.0 (ref)	0.9 (0.7−1.3)	1.0 (ref)

*Abbreviations*: *OA* osteoarthritis, *PY* person-years, *RD* rate difference, *HR* hazard ratio

## Discussion

This population-based cohort study, using a large sample of people with OA from an entire Canadian province, found that tramadol initiators were at an increased risk of mortality over the following year compared with initiators of naproxen (3.3 excess deaths attributable to tramadol per 1000 person-years), diclofenac (5.6 excess deaths attributable to tramadol per 1000 person-years), and Cox-2 inhibitors (8.1 excess deaths attributable to tramadol per 1000 person-years). Tramadol initiators were also at an increased risk of DVT (1.2 to 1.5 excess DVT events attributable to tramadol per 1000 person-years) and hip fractures (1.7 to 1.9 excess hip fractures attributable to tramadol per 1000 person-years) over the following year compared with all NSAIDs except naproxen. However, tramadol initiators did not have an increased risk of CVD over the following year compared with all NSAIDs. Furthermore, no difference among all outcomes was observed between tramadol and codeine cohorts.

Both tramadol and NSAIDs are commonly used pain-relief medications for OA patients. Recently, tramadol has been considered a potential alternative to NSAIDs because of its assumed lower risk of serious cardiovascular and gastrointestinal adverse effects than NSAIDs [11]. However, despite a few recently published population-based studies [11, 15, 16], comparisons of the safety profile of tramadol with other analgesics are limited. Our study used a truly population-based sample that includes data on all healthcare encounters and dispensed medications for all persons diagnosed with OA in BC. Our results are consistent with propensity score-matched cohort studies using the general practice data in the UK [11, 15, 16]. Results from those studies showed that among patients aged 50 years and older with OA, initial prescription of tramadol was associated with a 70–100% higher risk of mortality [11] and a 65–96% higher risk of hip fractures [15] over 1-year follow-up compared with commonly prescribed NSAIDs. However, there are some discrepant results between our study and previous studies. Specifically, the UK study found that the 180-day risk of incident MI among initiators of tramadol was higher compared with naproxen [16]. They have also shown that the initiation of tramadol was associated with a higher risk of hip fractures than the initiation of codeine [15]. Another propensity score-matched cohort study using the Medicare database in the USA found that the incidence of fractures was lower in tramadol initiators than that in codeine initiators among participants with a mean age of 80 years during the 180-day follow-up period [37]. Even though our study did not demonstrate an increased risk of CVD, given that CVD risk is already increased among NSAID users [38], a non-significant difference in risk between tramadol users and NSAID users may suggest an increased risk for tramadol use as compared to those not taking either of the medications. Xie et al. showed that tramadol was associated with a higher risk of all-cause mortality, compared with codeine [39]. Instead of focusing on OA patients, they investigated the association among the general population. Besides, the mean age of patients was 52.7 years in the tramadol cohort and 53.5 years in the codeine cohort, which is younger than patients in our study.

Several proposed explanations for the increased risk of mortality, VTE, and hip fractures among tramadol users compared to NSAID users exist: (1) tramadol may increase the postoperative delirium risk, which could potentially increase the risk of mortality [40]. (2) A higher risk of mortality might occur if patients take tramadol inappropriately, such as consuming alcohol or other central nervous system depressants while using tramadol [41]. (3) Tramadol might increase the coagulation of plasma proteins and inhibit the thrombocyte de-aggregation process which can increase the risk of VTE [42, 43]. (4) Tramadol may also lead to oxidative stress which has an important role in the development of atherosclerotic diseases [44, 45]. (5) Seizures [46], dizziness [47], and delirium [40] caused by tramadol could possibly increase the risk of falls which is one of the most common causes of hip fractures.

The limitations of our study deserve comment. Variables to measure OA disease severity are not available in our data. As such, confounding by indication is a potential issue in this study. Patients with more severe OA or contraindication for NSAIDs may be more likely to receive tramadol as compared to NSAIDs. While we attempted to control for confounding by indication by adjusting for propensity scores and were able to match on variables that are associated with OA pain (i.e., OA duration, comorbidities), we were unable to adjust for disease severity itself as this is not included in the administrative data. However, after the propensity score matching, all observed baseline variables were substantially balanced between comparison groups with all standardized differences less than 0.10. Second, physician-ordered dispensed medication may not reflect the actual medication use by patients and over-the-counter NSAID users may exist. Therefore, the misclassification of NSAID use could bias the results. However, all provinces in Canada have universal healthcare and PharmaNet data capture all outpatient dispensed medications for all residents. Third, the covariate OA duration may be subject to inaccuracy given that we can only capture healthcare services starting from 1990. Finally, although our sample size was large (*n* = 100,358), our outcomes of interest were rare, which affects the size of confidence intervals. Confidence intervals across comparison groups for a given outcome occasionally overlapped, but given the high prevalence of OA and common use of prescription medications for pain management in OA, even small differences in effect estimates are clinically important. Despite these limitations, there are several notable strengths. This is a population-based cohort study using a large Canadian administrative dataset that includes the entire population in a province and all dispensed drugs, making our results generalizable. The large sample size provided sufficient statistical power to study the safety profile of tramadol compared with commonly prescribed NSAIDs.

## Conclusions

Although further evidence on the relationship between tramadol and mortality and morbidity outcomes is required, the accumulation of evidence of the risks associated with its use suggests that current guidelines on tramadol use in clinical practice might need to be revisited. As there is no difference in pain relief between tramadol and NSAIDs among OA patients [13], the potential risk of VTE and hip fractures associated with tramadol use can further increase the burden of disease in patients already afflicted with moderate to severe OA. In addition, given that risks of CVD are already increased in NSAID users, an even non-statistically significant difference of the CVD risk compared between tramadol and NSAIDs can further demonstrate an unfavorable profile of tramadol use.

In conclusion, in this population-based cohort study, we found that the initiation of tramadol was associated with a higher risk of mortality (20–50%), VTE (70%), and hip fractures (40–60%) over 1 year of follow-up compared with commonly prescribed NSAIDs, but not with codeine.

## Supplementary Information

**Additional file 1: Supplemental Table 1**. Case definition of all-cause mortality, CVD, VTE, and hip fractures. Abbreviations: CVD, cardiovascular diseases; DVT, deep vein thrombosis; ICD-9, International Classification of Diseases, Ninth Revision; ICD-10, International Classification of Diseases, Tenth Revision; MI, myocardial infarction; PE, pulmonary embolism; VTE, venous thromboembolism.

**Additional file 2: Supplemental Figure 1**. Selection process of patients for the study.

**Additional file 3: Supplemental Material 1**. R code for additive hazard model.

## Data Availability

The data that support the findings of this study are available from Population Data BC, but restrictions apply to the availability of these data, which were used under license for the current study, and so are not publicly available.

## Abbreviations

BC: British Columbia
CI: Confidence interval
Cox-2: Cyclooxygenase-2
CVD: Cardiovascular diseases
DVT: Deep vein thrombosis
HR: Hazard ratio
MI: Myocardial infarction
NSAIDs: Nonsteroidal anti-inflammatory drugs
OA: Osteoarthritis
PE: Pulmonary embolism
RD: Rate difference
UK: United Kingdom
US: United States
VTE: Venous thromboembolism
