# Correction: Association of tramadol with all-cause mortality, cardiovascular diseases, venous thromboembolism, and hip fractures among patients with osteoarthritis: a population-based study

**DOI:** 10.1186/s13075-022-02805-x

**Published:** 2022-05-18

**Authors:** Lingyi Li, Shelby Marozoff, Na Lu, Hui Xie, Jacek A. Kopec, Jolanda Cibere, John M. Esdaile, J. Antonio Avina-Zubieta

**Affiliations:** 1 Arthritis Research Canada, 230-2238 Yukon Street, Vancouver, BC V5Y 3P2 Canada; 2 Experimental Medicine Program, Faculty of Medicine, University of British Columbia, Vancouver, Canada; 3 Faculty of Health Sciences, Simon Fraser University, Burnaby, Canada; 4 School of Population and Public Health, University of British Columbia, Vancouver, Canada; 5 Division of Rheumatology, Department of Medicine, University of British Columbia, Vancouver, Canada

**Correction: Arthritis Res Ther 24, 85 (2022)**

**https://doi.org/10.1186/s13075-022-02764-3**

Upon publication of the original article [1], it was noticed that final sentence in the Abstract's Results section was incorrect. The correct sentence is: "No differences were observed between tramadol and codeine for all events."

## References

[1] Li L, Marozoff S, Lu N (2022). Association of tramadol with all-cause mortality, cardiovascular diseases, venous thromboembolism, and hip fractures among patients with osteoarthritis: a population-based study. Arthritis Res Ther.

