# Supplementary material for: Association of tramadol with all-cause mortality, cardiovascular diseases, venous thromboembolism, and hip fractures among patients with osteoarthritis: a population-based study
Source: Arthritis Res Ther. 2022 Apr 11;24:85. doi: 10.1186/s13075-022-02764-3 (PMC8996663; doi:10.1186/s13075-022-02764-3)
Supplement: Supplementary file 1 — Additional file 1: Supplemental Table 1. Case definition of all-cause mortality, CVD, VTE, and hip fractures. Abbreviations: CVD, cardiovascular diseases; DVT, deep vein thrombosis; ICD-9, International Classification of Diseases, Ninth Revision; ICD-10, International Classification of Diseases, Tenth Revision; MI, myocardial infarction; PE, pulmonary embolism; VTE, venous thromboembolism. [file 13075_2022_2764_MOESM1_ESM.docx]

**Supplemental Table 1.** Case definition of all-cause mortality, CVD, VTE, and hip fractures

| Event | Case definition |
| --- | --- |
| All-cause mortality | Deaths from any cause during the follow-up period were derived from the vital statistics data. |
| CVD | For MI: diagnostic codes (ICD-9 codes: 410; ICD-10 codes: I21) in outpatient or hospitalization data in any position, or in vital statistics data as cause of death. For ischemic stroke: diagnostic codes (ICD-9 codes: 433,434; ICD-10 codes: I63-I66) in outpatient or hospitalization data in any position, or in vital statistics data as cause of death. |
| VTE | For PE: diagnostic codes (ICD-9: 415.1, 673.2, 639.6; ICD-10: O88.2, I26) in hospitalization data in any position or in vital statistics data as cause of death. For DVT: diagnostic codes (ICD-9: 453; ICD-10: I82.4, I82.9) from outpatient or hospitalization data in any position, or vital statistics data as cause of deaths. Incident PE or DVT outcomes were defined by a corresponding ICD code plus an outpatient prescription for any anticoagulant therapy (heparin, warfarin, or a similar agent) between one month before and six months after the ICD code date. Given that VTE is a potentially fatal disease, patients may have died before they received treatment; thus, for those patients who died within 2 months after a VTE, PE, or DVT diagnosis, anticoagulant therapy was not needed as part of the case definition. |
| Hip fractures | Diagnostic codes (ICD-9 codes: 820.0, 820.2; ICD-10 codes: S72.0, S72.1, S72.2) in hospitalization data in any position. |

**Abbreviations:** CVD, cardiovascular diseases; DVT, deep vein thrombosis; ICD-9, International Classification of Diseases, Ninth Revision; ICD-10, International Classification of Diseases, Tenth Revision; MI, myocardial infarction; PE, pulmonary embolism; VTE, venous thromboembolism.
