# Supplementary figures and images for: Association of tramadol with all-cause mortality, cardiovascular diseases, venous thromboembolism, and hip fractures among patients with osteoarthritis: a population-based study
Source: Arthritis Res Ther. 2022 Apr 11;24:85. doi: 10.1186/s13075-022-02764-3 (PMC8996663; doi:10.1186/s13075-022-02764-3)

**Supplemental Figure 1. Selection process of patients for the study**

**
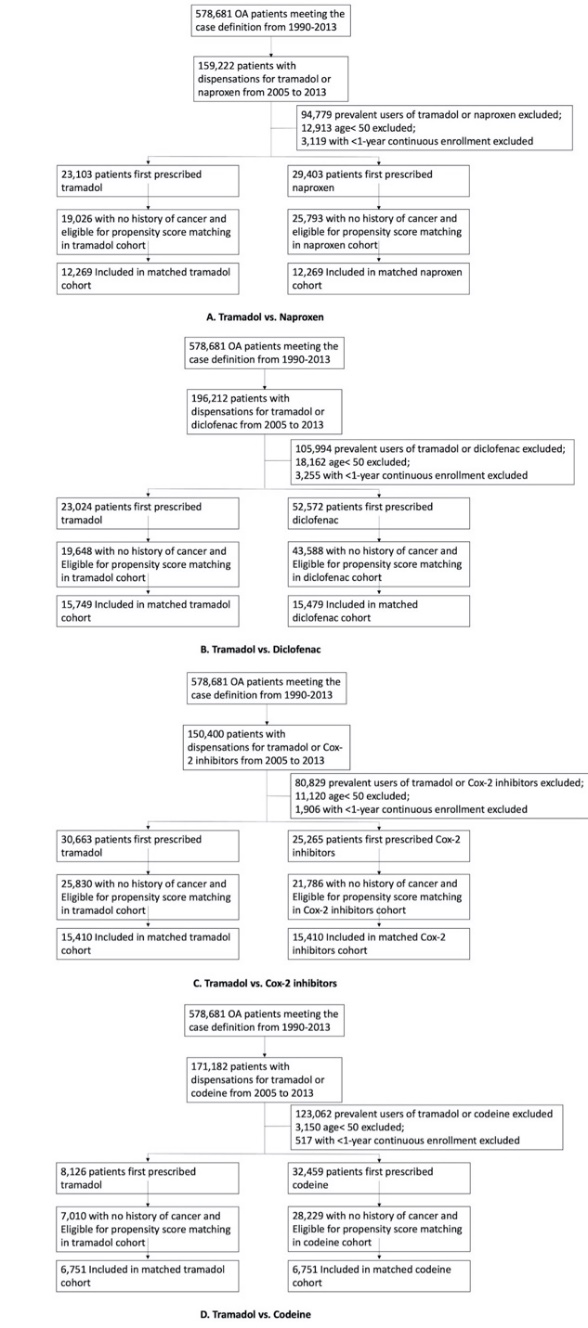
**

Supplement: Supplementary file 2 — Additional file 2: Supplemental Figure 1. Selection process of patients for the study. [file 13075_2022_2764_MOESM2_ESM.docx]
