# Supplementary material for: Association of tramadol with all-cause mortality, cardiovascular diseases, venous thromboembolism, and hip fractures among patients with osteoarthritis: a population-based study
Source: Arthritis Res Ther. 2022 Apr 11;24:85. doi: 10.1186/s13075-022-02764-3 (PMC8996663; doi:10.1186/s13075-022-02764-3)
Supplement: Supplementary file 3 — Additional file 3: Supplemental Material 1. R code for additive hazard model. [file 13075_2022_2764_MOESM3_ESM.docx]

**Supplemental Material 1. R code for additive hazard model**

R code for the additive hazard model:

#import PS matched data from SAS

library(haven)

DATA1 <- read_sas(“c:/…/data.sas7bdat”)

# Run additive hazard model

library(timereg)

fitAalen <- aalen(Surv(followup_time, event) ~ const(factor(EXPOSURE)), data=DATA1)

summary(fitAalen)
